# Effects of Supplementing Growing–Finishing Crossbred Pigs with Glycerin, Vitamin C and Niacinamide on Carcass Characteristics and Meat Quality

**DOI:** 10.3390/ani13233635

**Published:** 2023-11-24

**Authors:** Linglan Deng, Shaobin Hao, Wanjie Zou, Panting Wei, Wenchen Sun, Huadong Wu, Wei Lu, Yuyong He

**Affiliations:** 1 Jiangxi Province Key Laboratory of Animal Nutrition, Engineering Research Center of Feed Development, Jiangxi Agricultural University, Nanchang 330045, China; denglinglan2021@163.com (L.D.); haoshaobin1775@163.com (S.H.); zou19970812@163.com (W.Z.); 18679954991@163.com (P.W.); sun1221_wc@163.com (W.S.); lw20030508@163.com (W.L.); 2 College of Animal Science and Technology, Jiangxi Agricultural University, Nanchang 330045, China

**Keywords:** meat color, meat flavor, crossbred pig, fattening, glycerin, vitamins

## Abstract

**Simple Summary:**

Improving the color and flavor of pork from commercial crossbred pigs using nutritional measures is a major issue that needs to be urgently addressed. The objective of this study was to determine the influence of supplementing the diets of growing–finishing pigs with glycerin and/or a mixture of vitamin C and niacinamide on carcass traits and pork quality. The results showed that the simultaneous supplementation of growing–finishing crossbred pigs with glycerin, vitamin C and niacinamide can improve the color, flavor and nutritional value of pork by significantly increasing the redness (a*) value, glycerol content and myristoleic acid content of the *longissimus dorsi*; this is also achieved by numerically increasing the levels of flavor amino acids, linoleic acid, linolenic acid and erucic acid, as well as the percentage and density of type I myofibers in the *longissimus dorsi* and the *semimembranosus* muscle. Glycerin had a significant influence on the erucic acid content in the *longissimus dorsi* and the *semimembranosus* muscle, and the mixture of vitamin C and niacinamide had a significant interaction effect on the redness (a*) value of the *longissimus dorsi*. The glycerol content in the *longissimus dorsi* was significantly affected by glycerin, the interaction between vitamin C and niacinamide, and the interaction between glycerin, vitamin C and niacinamide. This study is valuable in improving pork quality and increasing consumers’ desire to purchase these products.

**Abstract:**

The objective of this study was to determine the influence of supplementing the diet of growing–finishing pigs with glycerin and/or a mixture of vitamin C and niacinamide on carcass traits and pork quality. Eighty-four weaned piglets with an initial average body weight of 20.35 ± 2.14 kg were assigned, at random, to four groups for a 103-day feeding experiment: control; glycerin-supplemented group; vitamin C and niacinamide-supplemented group; and glycerin, vitamin C and niacinamide-supplemented group. At the end of the experiment, three pigs/group were randomly selected and slaughtered, and samples were collected for analysis. The results indicated that supplementing crossbred pigs with glycerin, vitamin C and niacinamide simultaneously increased the redness (a*) value (*p* < 0.05), glycerol content (*p* < 0.01) and myristoleic acid content (*p* < 0.01) in the *longissimus dorsi* and tended to increase the level of flavor amino acids, linoleic acid, linolenic acid and erucic acid, as well as the percentage and density of type I myofibers in the *longissimus dorsi* and the *semimembranosus* muscle. Glycerin had an influence (*p* < 0.01) on the erucic acid content in the *longissimus dorsi* and the *semimembranosus* muscle, and vitamin C and niacinamide had an interaction effect (*p* < 0.05) on the redness (a*) value of the *longissimus dorsi*. Glycerin, vitamin C and niacinamide supplementation in the diet of crossbred pigs improved the color, flavor and nutritional value of pork, which contributed to an increased intent to purchase this product.

## 1. Introduction

Genetically improving crossbred pigs for a greater growth rate and lean percentage deteriorates their pork quality [1]; however, the consumer demand for pork with a bright red color, juiciness, tenderness and good flavor is increasing [2,3,4], and this is accompanied by an equally increasing demand for improved welfare conditions [5,6]. It is reported that the color of meat is generally controlled by deoxymyoglobin (DeoMb, purple-red), oxymyoglobin (OxyMb, bright red) and metmyoglobin (MetMb, brown), and can be improved by transforming MetMb into DeoMb and OxyMb via the action of enzymes or antioxidants such as vitamin C and niacinamide [7,8]. Nutritional regulation and feeding strategies are two effective means of improving pork quality [9,10,11]; thus, improving pork quality through nutritional means is a major issue that needs to be urgently addressed. 

The palatability of the feed is a significant factor that needs to be addressed, owing to the effect that the palatability of feed has on meat quality [12]. Glycerin is a sweet-tasting by-product of biodiesel production that has good palatability. It has attracted public attention because it is a viable alternative to corn, particularly owing to its high energy value and growing market supply [13,14]. Previous studies have verified the effect that adding crude glycerin to drinking water or employing it as a substitution for corn has on the performance, carcass characteristics and meat quality of pork; however, the results are still not consistent, as some data have indicated that a short-term glycerin supplementation of up to 15% in finishing pigs via drinking water or glycerin feeding has no negative effects on growth performance, carcass characteristics or pork quality [15,16,17,18]; meanwhile, other data have shown that the addition of glycerin to the diet of animals changes the fatty acid profile [19] or elevates the unsaturated fatty acid content [17,20,21] of meat. Vitamin C is capable of reducing the production of reactive oxygen species to protect cellular components such as lipids and proteins from damage and prolonging the reducing environment [22,23]. Adding vitamin C to the feed has been found to increase the concentration of vitamin C in muscle [24], while the treatment of meat with vitamin C solution has been found to decrease its brown metmyoglobin percentage and increase its bright red oxymyoglobin percentage [22,24]. Nicotinamide is a constituent of the nicotinamide adenine dinucleotide (NADH)-dependent MetMb reducing enzyme system, and a higher redness (a*) value and color stability in meat can be achieved by transforming MetMb into DeoMb and OxyMb under the action of NADH-dependent MetMb reductase [25,26].

The influence of supplementing the diets of growing–finishing crossbred pigs with glycerin, vitamin C and niacinamide on pork quality has still not been reported. We hypothesized that glycerin, vitamin C and niacinamide supplements would improve meat quality by increasing the marbling score and redness (a*) value of pork, and this study evaluated the effects of supplementing glycerin and/or a mixture of vitamin C and niacinamide on the carcass characteristics, chemical composition, amino acid composition and fatty acid profile of pork.

## 2. Materials and Methods

### 2.1. Animals, Basal Diet and Slaughter

A total of 84 weaned piglets (Duroc × Large White × Landrace) with initial body weights of 20.35 ± 2.14 kg were allocated to group A, group B, group C and group D at random using a 2 × 2 factorial design (glycerol: 0, 10; mixture of vitamin C and niacinamide: 0 + 0, 0.06 + 0.05) and offered the designated diets (Table 1). All piglets were housed in pens with concrete floors and had free access to food and water during the 103-day feeding experiment. At the end of the experiment, 3 pigs from each group were randomly selected, weighed after 12 h of fasting, slaughtered via bleeding after the application of electrical stimulus (225–380 V, 1.5 A, 5–6 s), and eviscerated. The final average live weights of the pigs slaughtered in groups A, B, C and D were 106.17 ± 8.39 kg, 106.00 ± 1.41 kg, 102.33 ± 10.21 kg and 109.17 ± 7.53 kg, respectively. 

Food-grade glycerin (purity > 99.5%) was purchased from Sichuan Lvcheng Biotechnology Co., Ltd. (Sichuan, China), and feed-grade Vitamin C (purity > 95.0%) and niacinamide (purity > 99.0%) were provided by Jiangxi Tianxin Pharmaceutical Co., Ltd. (Leping, China).

### 2.2. Blood Sampling and Serum Biochemical Parameters

Blood samples were taken from the anterior vena cava of the pigs on day 50 and day 101 of the experiment, respectively, and centrifuged for 10 min at 4 °C with a speed of 3000 r/min; then, the sample of serum was immediately collected and stored at −20 °C. The concentration of glucose (GLU) and lipids (total cholesterol, TC; triglyceride, TG; high-density lipoprotein, HDL; low-density lipoprotein, LDL; very-low-density lipoprotein, vLDL; non-esterified fatty acid, NEFA) in the serum was measured using an automatic biochemical analyzer (Mindray BS-240, Shenzhen Mindray Bio-Medical Electronics Co., Ltd., Shenzhen, China). A commercially available kit (HY-N0098, Beijing SINO-UK Institute of Biological Technology, Beijing, China) was used to measure the serum glycerol concentrations. The concentrations of transferrin (TF) and ferritin (FER) in the serum were determined using ELISA kits (Beijing SINO-UK Institute of Biological Technology, China), according to the manufacturer’s instructions.

### 2.3. Sample Collection, Carcass Characteristics and Meat Quality 

The carcass was divided into halves immediately after slaughter and the hot carcass weight was determined. The carcass length was measured on the left side of each carcass in a straight line, from the anterior edge of the first rib to the pubic bone, using a measuring tape. The back fat thickness was calculated by averaging the scores of three regions at the first rib, last rib and last lumbar vertebrae of the left carcass side. The back fat thickness was measured using a digital vernier caliper, between the 12th and 13th ribs over the *longissimus dorsi*, and averaged over three points. The loin muscle area was measured on the left side of the carcass using a compensating planimeter, after a cross-section cut was made between the 12th and 13th ribs; then, the muscle pH (pH 45 min; 45 min after slaughter) was measured using a Mettler M1120x pH meter (Mettler, Toledo International Inc., Columbus, OH, USA) in the center of the *longissimus dorsi* muscle. 

Marbling was measured in the *longissimus dorsi* between the 12th and 13th ribs using the scoring standards of the National Pork Producer Council. The meat color parameters L* (lightness), a* (redness) and b* (yellowness) were measured at four sites on each *longissimus dorsi* using a Minolta CR400 colorimeter (Konica Minolta, Osaka, Japan) 

Samples of the *longissimus dorsi* and the *semimembranosus* on the left side of the carcass were collected 1 h after the postmortem between the 12th and 13th ribs, then immediately refrigerated (4 °C) to analyze the chemical composition and the content of amino acids and fatty acids.

### 2.4. Nicotinamide Adenine Dinucleotide-Tetrazolium Reductase (NADH-TR) Staining [27]

The samples of muscle were embedded with an OCT complex (Sakura, Torrance, CA, USA), cross-sectioned into serial slices with a thickness of 10 μm using a HS-3060 cryostat microtome (Jinhua Hisure Technology Co., Ltd., Zhejiang, China), and then allowed to dry. After washing with PBS to remove the OCT complex, the slices were treated with the NADH-TR kit (Beijing SINO-UK Institute of Biological Technology, Beijing, China), according to the manufacturer’s instructions; then, myofibers of type I, IIa and IIb were stained dark blue, blue and light blue, respectively. 

Images of the slices were obtained using a color camera (IK-642K, Toshiba, Japan), six fields of view were randomly selected from each slice, and the image analysis system (Image-Pro Plus, Media Cybernetics, L.P., Rockville, MD, USA) was use d to analyze the percentage, diameter, cross-sectional area and density of the myofibers. 

### 2.5. Proximate Composition of Meat

The dry matter content was measured via freeze drying to a constant weight. The N content was determined using the Kjeldahl method described in AOAC (2006), and the crude protein was calculated as 6.25 × N. The crude fat was determined by extracting samples with petroleum ether in an automatic Soxhlet extractor (Gerhardt Analytical Systems, Königswinter, Germany). 

### 2.6. Content of Myoglobin and Glycerol in Muscle

The concentrations of myoglobin and glycerol in the *longissimus dorsi* and the *semimembranosus* were determined using ELISA kits (Shanghai Enzyme-linked Biotechnology Co., Ltd., Shanghai, China), according to the manufacturer’s instructions.

### 2.7. Amino Acids and Fatty Acid Profile

Freeze-dried muscle (about 1 g) was first ground, then hydrolyzed at 110 °C for 22 h in hydrochloric acid solution (15 mL, 6 mol/L). Further, the solution was diluted to 100 mL with water and centrifuged. The samples of supernatants were filtered with a 0.22 µm membrane prior to analysis using an automatic amino acid analyzer (L8800, Hitachi, Tokyo, Japan).

Approximately 6 g of freeze-dried muscle was ground, then cold-extracted with a solution of chloroform–methanol at a ratio of 2:1 to obtain lipids. The lipids were dried at 35 °C under vacuum conditions and then stored at −80 °C for the preparation of the fatty acid methyl esters (FAMEs). A gas chromatograph (Shimadzu GC-2010 Plus, Kyoto, Japan) equipped with a ZB-FAMEs GC capillary column (60 m * 0.25 mm * 0.2 μm, Phenomenex, Torrance, CA, USA) was used to separate and quantify the FAMEs.

The chromatographic conditions were as follows: an initial column temperature of 100 °C was held for 13 min, then increased at 10 °C/min increments to 180 °C for 6 min, then heated at 1 °C/min to 200 °C for 20 min, and then increased again at 4 °C/min increments up to a final temperature of 230 °C for 10.5 min. Nitrogen gas was used as the carrier gas at a constant flow rate of 0.9 mL/min. The split flow was 8 mL/min with a 1 μL injection volume. FAMEs were identified using authentic standards, and peak integration and quantification were performed with Shimadzu CLASS VP software (Version 4.3, Shimadzu, Kyoto, Japan). 

### 2.8. Statistical Analysis

SPSS 26.0 (IBM Inc., New York, NY, USA) software was utilized to perform the statistical analyses, the comparison of multiple groups was conducted using two-way ANOVA, and an LSD test was used to determine the significance of differences. All data were checked for normal distribution [28], and normally distributed data were expressed as means ± SEM; and differences were considered statistically significant when *p* < 0.05.

## 3. Results

### 3.1. Chemical Composition of Serum

The chemical composition of the serum is given in Table 2, and no statistical difference (*p* > 0.05) was observed in the serum concentration of the lipids, glucose, glycerol, transferrin and ferritin among the four treatment groups. The level of serum glycerol was influenced (*p* < 0.05) by the interaction between glycerin and the mixture of vitamin C and niacinamide, or by the mixture of vitamin C and niacinamide; the addition of glycerin had an impact (*p* < 0.05) on the TG concentration of the serum collected from pigs at d 101 of the experiment. Supplementing the growing–finishing pigs with the mixture of glycerin, vitamin C and niacinamide numerically decreased the serum glycerol concentration and increased the serum ferritin level when comparing the pigs in group D to those in group A; in addition, feeding glycerin to the growing–finishing pigs also numerically increased the serum ferritin concentration when comparing the pigs in group B to those in group A.

### 3.2. Carcass Characteristics, Meat Quality and Approximate Composition

Compared to the pigs in group A, supplementing the growing–finishing pigs with glycerin (group B), a mixture of vitamin C and niacinamide (group C), or a mixture of glycerin, vitamin C and niacinamide (group D) did not have a statistical influence (*p* > 0.05) on the carcass characteristics, marbling score, pH_45min_ and meat color of the pork (a*), except for the value associated with meat redness (Table 3). The *longissimus dorsi* of the finishing pigs in group D had a higher (*p* < 0.05) redness (a*) value than that of the finishing pigs in the other groups, and the addition of vitamin C and niacinamide had an influence (*p* < 0.05) on the redness (a*) value. The addition of glycerin, vitamin C and niacinamide tended to increase the carcass length and back fat thickness when comparing the pigs in group D to those in the other groups.

The results in Table 4 show that the addition of glycerin, a mixture of vitamin C and niacinamide, or a mixture of glycerin, vitamin C and niacinamide did not have a statistical influence (*p* > 0.05) on the content of moisture, crude protein, ether extract and myoglobin in the *longissimus dorsi* and the *semimembranosus* muscle, but that glycerin and the mixture of vitamin C and niacinamide had an interaction effect (*p* < 0.05) on the crude protein content of the *semimembranosus* muscle. The finishing pigs in group D had a numerically higher myoglobin level in the *longissimus dorsi* and the *semimembranosus* than the finishing pigs in the other groups. Supplementing the growing–finishing pigs with glycerin, a mixture of vitamin C and niacinamide, or a mixture of glycerin, vitamin C and niacinamide had different effects on the content of glycerol in the *longissimus dorsi* and *semimembranosus*; the finishing pigs in group B, group C and group D had a higher (*p* < 0.01) glycerol content in the *longissimus dorsi* than the finishing pigs in group A, but no statistical difference (*p* > 0.05) was found in the glycerol content of the *longissimus dorsi* among group B, group C and group D. In addition, the content of glycerol in the *longissimus dorsi* was affected by the glycerin (*p* < 0.05), the mixture of vitamin C and niacinamide (*p* < 0.01), and the interaction effect of glycerin and the mixture of vitamin C and niacinamide (*p* < 0.01). The finishing pigs in group C had a higher (*p* < 0.05) glycerol content in the *semimembranosus* than the finishing pigs in group A, group B and group D, but there was no statistical difference (*p* > 0.05) in the glycerol content of the *semimembranosus* in group A, group B and group D had, and the mixture of vitamin C and niacinamide had an influence (*p* < 0.05) on the content of glycerol in the *semimembranosus*. In addition, the *semimembranosus* muscle had a higher glycerol content than the *longissimus dorsi* muscle regardless of the treatment, with or without the supplementation of glycerin, vitamin C or niacinamide.

### 3.3. Composition and Characteristics of Myofibers

A higher (*p* < 0.01) percentage of type I myofibers, a lower (*p* < 0.01) percentage of type IIb myofibers, a smaller (*p* < 0.05) diameter and cross-sectional area of type IIb myofibers in the *longissimus dorsi* were observed when comparing the finishing pigs in group A to the finishing pigs in group B or group C (Table 5). There was no difference (*p* > 0.05) in the percentage, diameter, cross-sectional area and density of the myofibers in the *longissimus dorsi* when comparing the finishing pigs in group D to the finishing pigs in group A. The interaction effect between glycerin and the mixture of vitamin C and niacinamide had an influence on the percentage of myofibers (*p* < 0.01), the diameter of type IIb myofibers (*p* < 0.01), the cross-sectional area of type IIb myofibers (*p* < 0.01) and the density of type II myofibers (*p* < 0.05) in the *longissimus dorsi*.

The supplementation of the growing–finishing pigs with glycerin, a mixture of vitamin C and niacinamide, or a mixture of glycerin, vitamin C and niacinamide had no effect (*p* > 0.05) on the percentage of myofibers in the *semimembranosus* muscle when comparing the finishing pigs in group D to the finishing pigs in the other groups, but glycerin had an influence (*p* < 0.05) on the percentage of type I and IIb myofibers in the *semimembranosus* muscle. The addition of glycerin, vitamin C and niacinamide to the diets of the growing–finishing pigs tended to increase the percentage of type I and IIa myofibers, but tended to decrease the percentage of type IIb myofibers in the *semimembranosus* muscle. The addition of the vitamin C and niacinamide mixture increased (*p* < 0.05) the diameter and cross-sectional area of the myofibers in the *semimembranosus* when comparing the pigs in group C to the pigs in group A. The mixture of vitamin C and niacinamide had an effect (*p* < 0.05) on the diameter and cross-sectional area of the type I and IIa myofibers and the density of the type I myofibers in the *semimembranosus*.

### 3.4. Profiles of Amino Acids and Fatty Acids in Muscle

The amino acid profiles of muscle are shown in Table 6. The addition of glycerin, a mixture of vitamin C and niacinamide, or a mixture of glycerin, vitamin C and niacinamide to the diets of the growing–finishing pigs did not have a statistical effect (*p* > 0.05) on the level of amino acids in the *longissimus dorsi* and the *semimembranosus* muscle, but the addition of glycerin had an influence (*p* < 0.05) on the level of lysine, valine, phenylalanine, glutamate, aspartate, alanine, arginine, tyrosine and serine in the *longissimus dorsi* and on the level of glutamate, alanine, glycine and proline in the *semimembranosus;* in addition, the mixture of vitamin C and niacinamide had an effect (*p* < 0.05) on the methionine concentration of the *semimembranosus*. The addition of glycerin to the diets of the growing–finishing pigs numerically increased the level of all amino acids in the *longissimus dorsi* and the *semimembranosus* muscle when comparing the pigs in group B to the pigs in group A, group C and group D. The finishing pigs in group B and group D had a numerically higher (*p* > 0.05) content of flavor amino acids (glutamate, aspartate, alanine, glycine) in the *longissimus dorsi* and the *semimembranosus* muscle than the finishing pigs in group A.

Supplementing the growing–finishing pigs with glycerin, a mixture of vitamin C and niacinamide, a or mixture of glycerin, vitamin C and niacinamide had no statistical effect (*p* > 0.05) on the concentration of fatty acids in the *longissimus dorsi* and *semimembranosus*, except for C14:1n5 (myristoleic acid) and C22:1n9 (erucic acid) (Table 7); in addition, the finishing pigs in group D had a higher (*p* < 0.01) level of C14:1n5 in the *longissimus dorsi* than the finishing pigs in group A, but a lower (*p* < 0.05) level of C22:1n9 in the *semimembranosus* than the finishing pigs in group B. The addition of glycerin had an influence on the concentration of C14:1n5 (*p* < 0.01), C21:0 (*p* < 0.05) and C22:1n9 (*p* < 0.01) in the *longissimus dorsi*, and on the level of C22:1n9 (*p* < 0.01) in the *semimembranosus*. Glycerin and the mixture of vitamin C and niacinamide had an interaction effect on the level of C10:0 (*p* < 0.05), C12:0 (*p* < 0.05) and C14:1n5 (*p* < 0.01) in the *longissimus dorsi*, and on the level of C22:1n9 (*p* < 0.01) in the *semimembranosus* muscle. The finishing pigs in group D had a numerically higher content of C10:0, C12:0, C14:0, C14:1n5, C15:0, C16:0, C16:1n7, C17:0, C18:0, C18:1n9c, C18:2n6c, C18:3n3, C20:0, C20:1 and C20:2 in the *longissimus dorsi*, and a numerically higher content of C14:1n5, C16:1n7 and C22:6n3 in the *semimembranosus* than the finishing pigs in the other groups, respectively. The finishing pigs in group D had a higher (*p* > 0.05) level of essential fatty acids (linoleic acid, linolenic acid and arachidic acid) in the *longissimus dorsi* and the *semimembranosus* muscle than the finishing pigs in group A.

## 4. Discussion

The muscle color of finishing crossbred pigs is an important factor that influences the purchasing intent of consumers; this is because it is usually used to evaluate meat freshness [29,30], and generally consumers prefer meat with a pink color rather than a pale-white color. 

Previous studies found that the addition of glycerin has different influences on meat color, with crude glycerin feeding statistically decreasing the value of redness (a*) in the breast meat of Betong chicks [31] and in the *longissimus dorsi* of pigs [32], but statistically increasing the value of redness (a*) in beef [33]. In addition, the results of many other studies have also indicated that a diet with less than 2-% glycerin has no statistical influence on the redness (a*) value of pork [34,35], and only numerically increases the value of lightness, redness and yellowness in the meat of crossbred Boer goats [36]. Our study demonstrated that the addition of glycerin to the diet of pigs at 10% did not statistically increase the value of redness (a*) in pork, with growing–finishing pigs offered food-grade glycerin at 10% of the diet having a numerically lower redness (a*) value in the *longissimus dorsi* than pigs not offered food-grade glycerin in the diet; this is similar to previous reports [31,32]. However, the supplementation of vitamin C and niacinamide instead of glycerin numerically increased the value of redness (a*) in the *longissimus dorsi*; in addition, the growing–finishing pigs supplemented with glycerin, vitamin C and niacinamide simultaneously achieved a statistically higher value of redness (a*) in the *longissimus dorsi* than the pigs in the other groups. This result can be attributed to the highest content of OxyMb being present in the *longissimus dorsi* of group D. Myoglobin is the main pigment in muscle tissue, and it is classified into DeoMb (purple red), OxyMb (bright red) and MetMb (brown); the value of muscle redness (a*) depends on the amount and chemical state of myoglobin and the fatty acid composition of the myocyte membrane [8,26]. No statistical difference was observed in the myoglobin content among the four treatment groups, but group D had a statistically higher value of redness (a*) in the *longissimus dorsi* muscle than the other groups; this result might be attributed to the different chemical states of myoglobin. For the pigs in group D supplemented with vitamin C, niacinamide and glycerin, as well as those supplemented with vitamin C and niacinamide, antioxidant effects were observed. Meanwhile, glycerol has moisturizing effects and is able to reduce glycolysis; therefore, the content of oxygenated myoglobin in the *longissimus dorsi* of the pigs in group D should have been the highest. It has been reported that goats or ducks supplemented with vitamin C have a statistically higher redness (a*) than those not offered vitamin C [37,38]; this improvement in the redness value may be due to the ability of vitamin C to scavenge free radicals, inactivate reactive oxygen species, or improve the lipid stability by limiting the activities of reactive oxygen species [39]. 

Myofiber types also can influence the redness (a*) value of muscle [40,41]; these myofibers can be divided into oxidative types, including type I and IIa, which are red and rich in mitochondria, and into lipids, myoglobin and glycolytic type Iib, which are white and possess fewer mitochondria, lipids and myoglobin [42,43]. The supplementation of the diet of obese Zucker rats or growing pigs with niacin at 750 mg/kg induced type II myofiber to switch to type I muscle fiber, increasing the percentage of type I myofibers in the muscle [44,45]. Our study indicated that the percentage of type I and Iia myofibers in group C was lower than that in group A and B, respectively, but that the value of redness in the *longissimus dorsi* of group C was numerically higher than that in group A and B; this might be due to the increased reductase activity of metmyoglobin in the muscle after supplementation with nicotinamide, which resulted in more metmyoglobin being converted into oxymyoglobin [25,26]. Regarding the effect of glycerin, vitamin C or niacinamide on the development of myofibers, only a few previous studies have reported that treating the extensor carpi radialis longus and soleus muscles of rats with glycerin enlarges the cross-sectional area of fibers and increases the number of muscle fibers [46]; in another study, supplementing channel catfish with vitamin C at 50 mg/kg body weight increased the density and diameter of their myofibers [47], and the addition of vitamin C (250 mg/kg/day) and E (360 mg/kg) to rats statistically increased the cross-sectional area of the soleus and tibialis anterior compared to rats not treated with vitamin C and E [48]. In addition, an increase in the cross-sectional area and diameter of myofibers was observed in mice supplemented with nicotinamide or treated with nicotinamide N-methyltransferase [49,50]. At present, no studies addressing the influence of glycerin, vitamin C or niacinamide on the myofibers of pigs have been conducted, but our study indicated that growing–finishing pigs supplemented with glycerin or with a mixture of vitamin C and niacinamide had a larger diameter and cross-sectional area of myofibers than growing–finishing pigs not supplemented with glycerin, vitamin C and niacinamide; however, the underlying mechanism was not explored.

The fatty acids present in meat are important precursors of flavor compounds [51], and the majority of aromatic materials in meat are derived from lipids; in addition, the flavor characteristics of meat are strongly affected by the quantity of unsaturated fatty acids [52]. Previous studies have shown that the inclusion of glycerin in the diet of crossbred bulls statistically decreases the total saturated fatty acids and increases the content of MUFA and PUFA [20]; in addition, it has been found that the content of capric, lauric, myristic, palmitic, stearic and palmitoleic linearly decreases, but the content of pentadecanoic, heptadecanoic, transvaccenic, oleic, linolenic fatty acids, MUFA and PUFA increases when crude glycerin is included in the diet of lambs [53]. Pork presents with a predominance of oleic, palmitic, linoleic, stearic and arachidonic acid [54]. In their study, Faria et al. reported that the addition of crude glycerin at 5, 10, 15 and 20% to the diets of growing–finishing pigs did not statistically increase the content of fatty acids in the *longissimus dorsi* and the *semimembranosus* [55]; our study also did not find statistical differences in the fatty acid profile of the *longissimus dorsi* and the *semimembranosus* muscle among the four groups, except for myristoleic and erucic acid. However, a higher concentration of myristoleic acid, erucic acid and essential fatty acids (linoleic acid and linolenic acid) was achieved in the *longissimus dorsi* and the *semimembranosus* muscle of pigs supplemented simultaneously with glycerin, vitamin C and niacinamide, and in the *semimembranosus* muscle of pigs supplemented singly with glycerin; this is compared to that of pigs not supplemented with glycerin, vitamin C and niacinamide. The consumption of meat with high concentrations of myristoleic acid and essential fatty acids (linoleic and linolenic) is seen to be highly beneficial to human health; this is because reports have indicated that myristoleic acid can reduce adiposity [56], improve hyperglycemia [57] and alleviate inflammation [58]. Interestingly, our results indicated that supplementing the diet of pigs with glycerin alongside a rapeseed meal statistically increased the accumulation of erucic acid in the *longissimus dorsi* and the *semimembranosus* muscle, but that the singular addition of vitamin C and niacinamide did not present this accumulative effect. To date, the possible mechanism of action implicated in the accumulation of erucic acid in muscle with the addition of glycerin remains unclear. Erucic acid is a controversial fatty acid, as excessive erucic acid may generate the risk of intrauterine growth retardation [59], as well as induce hepatic steatosis and myocardial lipidosis [60]; however, other studies have found that erucic acid has antiviral, anti-inflammatory and anti-oxidative functions [61,62], and can act as a neuroprotective, anti-tumor and myelin protective agent [63]. 

No statistical difference was observed in the redness (a*) value of the *longissimus dorsi* between group B and group C, but the redness value of the *longissimus dorsi* in group D was statistically higher than that in group B and group C; this might be attributed to the significant interaction effect between glycerin and the mixture of vitamin C and niacinamide. In addition, the statistically significant increase in the percentage of type I myofibers and myristoleic acid content of the *longissimus dorsi* of pigs supplemented with a mixture of glycerin, vitamin C and niacinamide may also be ascribed to the interaction between glycerin and the mixture of vitamin C and niacinamide. This is because the statistical results indicated that this interaction had a statistically significant impact on the percentage of type I myofibers and the myristoleic acid content. However, no results regarding the effect of the interaction between glycerin and the mixture of vitamin C and niacinamide on the redness (a*) value, percentage of type I myofibers and myristoleic acid content of meat were found to support our hypothesis. 

In this study, we investigated the effect of supplementing the diet of pigs with glycerin and/or a mixture of vitamin C and niacinamide on pork quality, although the preliminary results indicated that supplementing the diet of growing–finishing pigs with glycerin, vitamin C and niacinamide together during a 103-day feeding experiment statistically increased the redness (a*) value, the percentage of type I myofibers and the myristoleic acid content of the *longissimus dorsi*. However, the limited size of the pigs slaughtered might have affected the generalizability of this work; for this reason, further studies with a larger sample size are needed to verify the findings of this study. 

## 5. Conclusions

Compared to the diet of growing–finishing crossbred pigs without the supplementation of glycerin, vitamin C and niacinamide, the diet supplemented with a mixture of glycerin, vitamin C and niacinamide significantly increased the redness (a*) value, glycerol and myristoleic acid content of the *longissimus dorsi*; tended to increase the level of flavor amino acids, linoleic acid, linolenic acid and erucic acid; and increased the percentage and density of type I myofibers in the *longissimus dorsi* and the *semimembranosus* muscle. Vitamin C and niacinamide had a significant interaction effect on the redness value of the *longissimus dorsi*, and the glycerol content of the *longissimus dorsi* was significantly affected by glycerin, the mixture of vitamin C and niacinamide, and the interaction of glycerin, vitamin C and niacinamide. Glycerin had a significant influence on the erucic acid content of the *longissimus dorsi* and the *semimembranosus* muscle. The underlying mechanism of action implicated in the increased value of redness (a*) and content of glycerol and myristoleic acid of the *longissimus dorsi* via the addition of glycerin, vitamin C and niacinamide should be further explored. In addition, the findings of this study will contribute to the production of high-quality pork that satisfies consumers’ purchase desires.

## Figures and Tables

**Table 1:** Diet composition and nutrient level (%, on dry matter basis).

	Group A		Group B		Group C		Group D	
	10–60 kg	60–120 kg	10–60 kg	60–120 kg	10–60 kg	60–120 kg	10–60 kg	60–120 kg
Ingredients								
Corn	59.00	64.00	48.00	52.00	58.89	63.89	47.89	51.89
Wheat bran	16.00	16.00	15.00	15.00	16.00	16.00	15.00	15.00
Glycerin	0.00	0.00	10.00	10.00	0.00	0.00	10.00	10.00
Soybean meal	14.00	10.00	15.00	11.00	14.00	10.00	15.00	11.00
Fishmeal	2.00	0.00	2.00	0.00	2.00	0.00	2.00	0.00
Rapeseed meal	5.00	6.00	6.00	8.00	5.00	6.00	6.00	8.00
4% Premix ^(1)^	4.00	4.00	4.00	4.00	4.00	4.00	4.00	4.00
Vitamin C	0.00	0.00	0.00	0.00	0.06	0.06	0.06	0.06
Niacinamide	0.00	0.00	0.00	0.00	0.05	0.05	0.05	0.05
Total	100.00	100.00	100.00	100.00	100.00	100.00	100.00	100.00
Nutrient levels ^(2)^								
ME (MJ/kg)	12.15	12.24	12.26	12.31	12.15	12.24	12.26	12.31
Crude protein	17.30	15.10	17.34	15.18	17.22	15.05	17.36	15.21
Crude fiber	3.84	3.68	3.69	3.78	3.74	3.71	3.47	3.63
Ether extract	3.81	3.68	3.43	3.50	3.94	3.88	3.57	3.65
Calcium	0.79	0.65	0.82	0.70	0.73	0.69	0.78	0.63
Total phosphorus	0.67	0.54	0.63	0.55	0.69	0.59	0.66	0.60
Lysine	1.09	0.84	1.09	0.86	1.09	0.84	1.09	0.86
Methionine + Cystine	0.57	0.44	0.56	0.43	0.57	0.44	0.56	0.43

Note: (1) The premix provided the following per kg of the diet in the 10 to 60 kg stage: VA, 2750 IU; VD3, 500 IU; VE, 11 IU; VK3, 0.50 mg; VB1, 0.55 mg; VB2, 1.55 mg; VB6, 0.80 mg; VB12, 10 µg; Niacotinic acid, 7.5 mg; pantothenic acid, 6.5 mg; biotin, 0.08 mg; Cu (CuSO_4_·5H_2_O), 5 mg; Zn (ZnSO_4_·1H_2_O), 17.5 mg; Mn (MnSO_4_·1H_2_O), 5 mg; Fe (FeSO_4_·1H_2_O), 21.3 mg; Se (Na_2_SeO_3_), 0.07 mg; I (KI), 0.08 mg; Co(CoCl_2_·1H_2_O), 0.06 mg; Lysine, 90 g; Methionine, 71 g. The premix provided the following per kg of the diet in the 60 to 90 kg stage: lys, 90 g; VA, 1600 IU; VD3, 300 IU; VE, 11 IU; VK3, 0.50 mg; VB1, 0.48 mg; VB2 1.10, mg; VB6, 0.65 mg; VB12, 9 µg; Niacotinic acid, 6.5 mg; pantothenic acid, 6.0 mg; biotin, 0.06 mg; Cu (CuSO_4_·5H_2_O), 5 mg; Zn (ZnSO_4_·1H_2_O), 13.5 mg; Mn (MnSO_4_·1H_2_O), 5 mg; Fe (FeSO_4_·1H_2_O), 17.0 mg; Se (Na_2_SeO_3_), 0.07 mg; I (KI), 0.08 mg; Co(CoCl_2_·1H_2_O), 0.06 mg; lysine, 69 g; methionine, 49 g. (2) Metabolizable energy (ME), lysine, methionine and cystine are calculated values. Crude protein, crude fiber, ether extract, calcium and total phosphorus are measured values.

**Table 2:** Comparison of serum chemical parameters.

	Group A	Group B	Group C	Group D	SEM	*p*-Value		
						GLY	VC + NM	GLY × (VC + NM)
Serum of pigs sampled at day 50 of the experiment								
TC (mmol/L)	1.73	1.88	1.62	1.62	0.13	0.590	0.208	0.587
TG (mmol/L)	0.28	0.30	0.27	0.22	0.03	0.615	0.181	0.218
HDL (mmol/L)	0.70	0.65	0.59	0.61	0.05	0.801	0.184	0.468
LDL (mmol/L)	0.90	1.02	0.88	0.80	0.09	0.794	0.229	0.320
vLDL (mmol/L)	0.48	0.54	0.49	0.35	0.05	0.417	0.067	0.060
NEFA (mmol/L)	0.07	0.06	0.05	0.07	0.01	0.535	0.713	0.207
GLU (mmol/L)	4.32	4.46	4.58	3.58	0.28	0.162	0.304	0.074
GL (umol/L)	3.58	3.74	4.17	2.37	0.39	0.067	0.339	0.036
TF (g/L)	2.82	2.73	2.88	2.57	0.19	0.314	0.793	0.567
FER (ng/mL)	90.90	103.87	89.62	98.23	10.01	0.312	0.739	0.833
Serum of pigs sampled at day 101 of the experiment								
TC (mmol/L)	1.88	1.67	1.82	1.79	0.22	0.598	0.900	0.718
TG (mmol/L)	0.35	0.28	0.36	0.32	0.02	0.042	0.302	0.469
HDL (mmol/L)	0.62	0.55	0.62	0.58	0.10	0.616	0.861	0.888
LDL (mmol/L)	1.02	0.97	1.03	0.98	0.14	0.742	0.971	0.994
vLDL (mmol/L)	0.55	0.49	0.55	0.54	0.07	0.635	0.748	0.705
NEFA (mmol/L)	0.08	0.08	0.37	0.55	0.24	0.700	0.146	0.704
GLU (mmol/L)	4.58	5.36	5.42	4.53	0.66	0.938	0.991	0.241
GL (umol/L)	5.49	5.40	4.56	3.50	0.49	0.275	0.021	0.357
TF (g/L)	2.61	2.82	2.63	2.64	0.23	0.630	0.722	0.661
FER (ng/mL)	93.60	110.10	94.82	95.52	6.04	0.193	0.301	0.227

Note: TC, total cholesterol; TG, triglyceride; HDL, high-density lipoprotein; LDL, low-density lipoprotein; vLDL, very-low-density lipoprotein; NEFA, non-esterified fatty acid; GLU, glucose; GL, glycerol; TF, transferrin; FER, ferritin; GLY, glycerin; VC, vitamin C; NM, niacinamide. Means within a row followed by no or the same letters do not differ significantly (*p* > 0.05). *p*-value means the effect of GLY, (VC + NM), or the interaction of GLY and (VC + NM) on the parameter within the same row.

**Table 3:** Carcass characteristics, marbling score, pH and meat color.

	Group A	Group B	Group C	Group D	SEM	*p*-Value		
						GLY	VC + NM	GLY × (VC + NM)
Carcass characteristics								
Carcass weight (kg)	38.50	39.28	37.00	38.67	2.11	0.578	0.630	0.839
Carcass length (cm)	75.67	75.33	72.00	79.33	2.31	0.168	0.944	0.136
Backfat thickness (cm)	12.00	13.35	13.21	14.03	3.15	0.738	0.772	0.934
Loin muscle area (mm^2^)	4014.11	4232.07	3669.58	4040.00	306.29	0.365	0.407	0.810
Longissimus dorsi								
Marbling score	2.00	2.00	1.83	2.00	0.53	0.878	0.878	0.878
pH_45 min_	6.23	6.33	6.08	6.28	0.18	0.452	0.595	0.786
L* (lightness)	42.17	42.66	42.80	42.30	1.76	0.997	0.940	0.786
a* (redness)	1.86 ^b^	1.22 ^b^	2.94 ^b^	4.79 ^a^	0.62	0.352	0.011	0.090
b* (yellowness)	9.48	9.78	9.93	9.86	0.50	0.820	0.616	0.721

Means within a row followed by different lowercase letters differ significantly (*p* < 0.05). *p*-value means the effect of GLY, (VC + NM) or the interaction of GLY and (VC + NM) on the parameter within the same row.

**Table 4:** Comparison of proximate composition (%, as fresh basis).

	Group A	Group B	Group C	Group D	SEM	*p*-Value		
						GLY	VC + NM	GLY × (VC + NM)
Longissimus dorsi								
Moisture	72.28	73.20	73.62	73.35	0.66	0.633	0.291	0.389
Crude protein	20.27	20.60	20.16	19.78	0.27	0.929	0.120	0.218
Ether extract	4.88	4.30	4.16	4.81	0.62	0.954	0.862	0.354
Myoglobin (ng/mL)	9.23	8.63	8.27	9.27	0.52	0.706	0.763	0.163
Glycerol (µmol/g)	2.84 ^B^	4.23 ^A^	4.48 ^A^	4.56 ^A^	0.23	0.013	0.003	0.022
Semimembranosus								
Moisture	72.91	72.84	72.80	72.50	0.35	0.606	0.529	0.750
Crude protein	19.96	19.31	19.65	20.19	0.22	0.791	0.231	0.028
Ether extract	5.43	5.69	5.43	5.53	0.41	0.674	0.851	0.844
Myoglobin (ng/mL)	7.90	8.27	8.27	8.50	0.88	0.739	0.745	0.936
Glycerol (μmol/g)	5.39 ^b^	5.32 ^b^	7.53 ^a^	5.46 ^b^	0.47	0.051	0.040	0.064

Means within a row followed by different lowercase letters differ significantly (*p* < 0.05) and those followed by different uppercase letters differ significantly (*p* < 0.01). *p*-value means the effect of GLY, (VC + NM), or the interaction of GLY and (VC + NM) on the parameter within the same row.

**Table 5:** A comparison of the different types of myofibers among the different groups.

		Group A	Group B	Group C	Group D	SEM	*p*-Value		
							GLY	VC + NM	GLY × (VC + NM)
Longissimus dorsi									
Percentage (%)	Type I	18.77 ^AB^	14.17 ^BC^	12.09 ^C^	20.91 ^A^	1.46	0.185	0.984	0.002
	Type II a	33.46 ^a^	26.05 ^b^	27.05 ^b^	29.52 ^ab^	1.45	0.126	0.340	0.009
	Type II b	47.78 ^B^	59.78 ^A^	60.86 ^A^	49.57 ^B^	2.37	0.884	0.560	0.001
Diameter (μm)	Type I	44.90	51.91	48.70	45.40	3.73	0.633	0.726	0.204
	Type II a	53.43	61.01	64.01	47.71	5.71	0.467	0.817	0.070
	Type II b	73.91 ^b^	106.64 ^a^	111.39 ^a^	72.09 ^b^	9.63	0.741	0.883	0.006
Cross-sectional area (μm^2^)	Type I	2062.67	2697.15	2395.67	2084.38	339.92	0.647	0.691	0.202
	Type II a	2848.92	3812.71	4195.70	2312.01	681.08	0.519	0.913	0.070
	Type II b	5661.33 ^bc^	11,508.86 ^ab^	12,704.35 ^a^	5322.78 ^c^	1805.55	0.682	0.818	0.006
Density (number/μm^2^)	Type I	37.29	53.74	50.48	28.09	9.32	0.758	0.522	0.071
	Type II a	87.21	155.28	141.17	72.57	23.83	0.991	0.563	0.021
	Type II b	117.34	208.94	189.95	97.65	32.07	0.991	0.563	0.021
	Total	241.84	417.96	381.60	198.30	65.05	0.957	0.556	0.025
Semimembranosus									
Percentage (%)	Type I	17.96	22.94	18.00	24.65	2.48	0.047	0.733	0.744
	Type II a	29.15	30.22	28.16	32.19	1.23	0.071	0.701	0.264
	Type II b	52.89	46.84	53.84	43.16	3.52	0.045	0.708	0.529
Diameter (μm)	Type I	43.86 ^b^	51.19 ^ab^	64.76 ^a^	57.16 ^ab^	4.02	0.974	0.010	0.100
	Type II a	51.41 ^b^	52.27 ^b^	73.20 ^a^	58.65 ^ab^	4.88	0.198	0.020	0.153
	Type II b	81.68	75.94	117.42	78.87	9.99	0.058	0.089	0.139
Cross-sectional area (μm^2^)	Type I	1987.81 ^b^	2775.58 ^b^	4590.26 ^a^	3453.98 ^ab^	509.64	0.741	0.012	0.096
	Type II a	2709.25 ^b^	2765.22 ^b^	5451.16 ^a^	3457.57 ^ab^	624.85	0.160	0.025	0.140
	Type II b	7078.73 ^b^	5890.88 ^b^	14,012.35 ^a^	6276.35 ^b^	1860.85	0.043	0.085	0.117
Density (number/μm^2^)	Type I	39.26	35.23	70.66	44.24	8.49	0.111	0.045	0.224
	Type II a	104.44	89.71	190.95	104.76	23.68	0.066	0.064	0.170
	Type II b	140.53	120.72	256.94	140.96	31.87	0.066	0.064	0.170
	Total	284.23	245.66	518.55	289.95	63.90	0.070	0.061	0.175

Means within a row followed by different lowercase letters differ significantly (*p* < 0.05), and those followed by different uppercase letters differ significantly (*p* < 0.01). *p*-value means the effect of GLY, (VC + NM), or the interaction of GLY and (VC + NM) on the parameter within the same row.

**Table 6:** Amino acid composition of muscle (g/100 g, on fresh basis).

	Group A	Group B	Group C	Group D	SEM	*p*-Value		
						GLY	VC + NM	GLY × (VC + NM)
Longissimus dorsi								
Essential amino acids (EAAs)								
Lysine	1.94	2.08	1.96	2.03	0.03	0.014	0.708	0.317
Methionine	0.42	0.47	0.43	0.42	0.03	0.400	0.463	0.293
Valine	1.10	1.16	1.10	1.13	0.02	0.037	0.337	0.428
Leucine	1.73	1.82	1.75	1.79	0.03	0.086	0.884	0.419
Isoleucine	1.03	1.07	1.03	1.04	0.03	0.461	0.620	0.620
Phenylalanine	0.86	0.92	0.87	0.90	0.02	0.022	0.676	0.411
Histidine	1.04	1.03	1.02	1.00	0.02	0.585	0.204	0.695
Threonine	0.95	1.01	0.95	0.97	0.02	0.051	0.246	0.328
Non-essential amino acids (NEAAs)								
Glutamate	3.20	3.43	3.21	3.33	0.07	0.032	0.532	0.447
Aspartate	2.02	2.15	2.06	2.11	0.03	0.019	1.000	0.283
Alanine	1.22	1.30	1.23	1.25	0.02	0.040	0.406	0.198
Arginine	1.32	1.42	1.34	1.38	0.03	0.022	0.803	0.234
Glycine	0.89	0.95	0.92	0.90	0.02	0.361	0.616	0.125
Tyrosine	0.71	0.77	0.72	0.74	0.02	0.036	0.600	0.264
Serine	0.80	0.85	0.81	0.83	0.01	0.031	0.719	0.411
Proline	0.63	0.66	0.62	0.61	0.01	0.608	0.065	0.147
FAAs ^1^	8.91	9.52	9.01	9.25	0.154	0.026	0.582	0.256
Total EAAs	9.07	9.57	9.11	9.28	0.15	0.061	0.428	0.313
Total NEAAs	10.80	11.53	10.92	11.17	0.18	0.027	0.530	0.226
TAAs	19.87	21.10	20.03	20.45	0.33	0.036	0.473	0.253
Semimembranosus								
Essential amino acids (EAAs)								
Lysine	1.94	2.01	1.81	1.94	0.06	0.113	0.113	0.663
Methionine	0.39	0.41	0.29	0.34	0.03	0.213	0.018	0.636
Valine	1.09	1.11	1.02	1.09	0.03	0.099	0.099	0.363
Leucine	1.74	1.79	1.62	1.74	0.05	0.128	0.128	0.559
Isoleucine	1.02	1.06	0.96	1.01	0.03	0.170	0.105	0.835
Phenylalanine	0.87	0.90	0.81	0.88	0.02	0.080	0.151	0.552
Histidine	0.91	0.97	0.89	0.93	0.03	0.120	0.327	0.737
Threonine	0.93	1.01	0.91	0.96	0.03	0.054	0.193	0.648
Non-essential amino acids (NEAAs)								
Glutamate	3.21	3.57	3.17	3.36	0.10	0.027	0.255	0.394
Aspartate	2.04	2.13	1.93	2.07	0.06	0.065	0.149	0.664
Alanine	1.21	1.26	1.16	1.25	0.03	0.042	0.273	0.554
Arginine	1.31	1.38	1.23	1.30	0.04	0.104	0.060	0.933
Glycine	0.88	0.97	0.88	0.95	0.03	0.020	0.863	0.689
Tyrosine	0.71	0.75	0.66	0.72	0.02	0.066	0.130	0.831
Serine	0.80	0.85	0.78	0.83	0.02	0.070	0.358	1.000
Proline	0.60	0.66	0.60	0.66	0.02	0.010	0.921	0.921
FAAs ^1^	8.91	9.59	8.62	9.22	0.243	0.029	0.212	0.873
Total EAAs	8.88	9.27	8.32	8.88	0.26	0.104	0.104	0.733
Total NEAAs	10.76	11.58	10.41	11.13	0.29	0.029	0.205	0.859
TAAs	19.64	20.85	18.73	20.01	0.55	0.052	0.148	0.946

^1^ FAAs = glutamate + aspartate + phenylalanine + alanine + glycine + tyrosine. Means within a row followed by no letters or the same letters do not differ significantly (*p* > 0.05). *p*-value means the effect of GLY, (VC + NM), or the interaction of GLY and (VC + NM) on the parameter within the same row. *p*-value means the effect of GLY, (VC + NM), or the interaction of GLY and (VC + NM) on the parameter within the same row.

**Table 7:** Fatty acid profiles of muscle (mg/kg, on fresh basis).

	Group A	Group B	Group C	Group D	SEM	*p*-Value		
						GLY	VC + NM	GLY × (VC + NM)
Longissimus dorsi								
C10:0 (Capric)	19.27	16.83	15.83	23.23	1.86	0.218	0.447	0.029
C12:0 (Lauric)	18.23	17.00	13.93	20.23	1.63	0.159	0.752	0.050
C14:0 (Myristic)	352.13	320.47	282.47	396.33	40.90	0.344	0.941	0.113
C14:1n5 (Myristoleic)	3.27 ^BC^	3.50 ^B^	2.67 ^C^	4.40 ^A^	0.22	0.002	0.510	0.009
C15:0 (Pentadecanoic)	5.30	6.30	5.33	8.60	1.30	0.140	0.397	0.410
C16:0 (Palmitic)	9694.67	8987.13	7774.73	10,636.03	1205.77	0.398	0.913	0.177
C16:1n7 (Palmitoleic)	941.30	893.53	804.27	1186.80	94.79	0.115	0.434	0.053
C17:0 (Heptadecanoic)	36.80	41.73	32.30	54.13	10.52	0.239	0.717	0.445
C18:0 (Stearic)	4876.93	4620.83	3780.97	5277.70	695.90	0.399	0.760	0.243
C18:1n9c (Oleic)	19,711.23	16,653.53	16,018.43	20,815.17	1892.72	0.658	0.904	0.072
C18:2n6c (Linoleic)	1667.83	1465.80	1630.33	2136.83	254.43	0.566	0.248	0.201
C18:3n3 (Linolenic)	67.60	62.80	64.20	98.60	12.26	0.262	0.223	0.149
C20:0 (Arachidic)	60.53	63.40	51.00	73.80	10.04	0.237	0.967	0.350
C20:1 (Cis-11-eicosenoic)	298.27	253.43	251.33	324.33	40.27	0.736	0.774	0.182
C20:2 (Eicosadienoic)	69.73	59.53	71.43	86.90	10.38	0.806	0.199	0.252
C20:3n6 (Eicosatrienoic)	20.03	21.40	23.07	21.13	3.03	0.928	0.660	0.601
C20:4n6 (Arachidonic)	120.63	133.23	120.60	120.80	7.35	0.409	0.421	0.424
C20:5n3 (Eicosapentaenoic)	5.63	5.40	6.53	5.27	0.97	0.463	0.704	0.610
C21:0 (Heneicosanoic)	7.67	11.03	7.07	10.70	1.24	0.023	0.717	0.917
C22:1n9 (Erucic)	10.13 ^b^	18.87 ^a^	10.27 ^b^	16.07 ^ab^	2.04	0.007	0.531	0.492
C22:6n3 (Docosahexaenoic)	13.33	11.07	33.40	15.57	7.35	0.209	0.133	0.321
SFAs ^1^	15,071.53	14,084.73	11,963.63	16,500.77	1951.54	0.390	0.864	0.195
MUFAs ^2^	20,964.20	17,822.87	17,086.97	22,346.77	2000.25	0.611	0.876	0.069
PUFAs ^3^	1964.80	1759.23	1949.57	2485.10	282.23	0.575	0.244	0.226
n-3 PUFAs ^4^	86.57	79.27	104.13	119.43	15.22	0.799	0.094	0.479
n-6 PUFAs ^5^	1808.50	1620.43	1774.00	2278.77	258.94	0.558	0.263	0.218
USFAs	22,929.00	19,582.10	19,036.53	24,831.87	2175.79	0.589	0.763	0.069
TFAs	38,000.53	33,666.83	31,000.17	41,332.63	4089.25	0.484	0.937	0.111
Semimembranosus								
C10:0 (Capric)	19.23	21.60	20.40	21.33	2.87	0.581	0.879	0.809
C12:0 (Lauric)	16.93	18.97	28.10	19.53	6.54	0.631	0.396	0.441
C14:0 (Myristic)	341.83	396.73	390.63	380.53	58.07	0.710	0.786	0.591
C14:1n5 (Myristoleic)	2.83	3.80	3.67	4.03	0.87	0.464	0.556	0.739
C15:0 (Pentadecanoic)	8.80	9.67	12.83	10.20	3.05	0.779	0.475	0.582
C16:0 (Palmitic)	9787.00	10,348.50	9587.60	9899.37	1331.55	0.751	0.814	0.928
C16:1n7 (Palmitoleic)	897.70	1123.37	918.23	1160.80	148.21	0.153	0.850	0.956
C17:0 (Heptadecanoic)	51.73	61.63	56.57	59.17	8.90	0.503	0.898	0.693
C18:0 (Stearic)	4953.00	5066.10	4327.30	4635.80	605.28	0.737	0.408	0.876
C18:1n9c (Oleic)	20,544.80	21,396.20	19,421.10	20,323.67	2274.69	0.710	0.642	0.991
C18:2n6c (Linoleic)	2786.83	3201.47	3063.53	2891.93	219.79	0.595	0.942	0.219
C18:3n3 (Linolenic)	107.80	152.63	123.67	129.23	13.23	0.093	0.783	0.176
C20:0 (Arachidic)	60.20	74.03	67.83	65.67	12.32	0.648	0.977	0.534
C20:1 (Cis-11-eicosenoic)	312.83	356.20	295.70	329.80	44.75	0.412	0.640	0.920
C20:2 (Eicosadienoic)	113.87	116.73	119.03	112.37	9.57	0.848	0.968	0.632
C20:3n6 (Eicosatrienoic)	31.83	34.83	27.70	29.90	2.20	0.271	0.073	0.860
C20:4n6 (Arachidonic)	227.03	195.43	149.23	188.00	23.71	0.884	0.110	0.176
C20:5n3 (Eicosapentaenoic)	11.97	14.90	6.90	10.50	3.28	0.349	0.187	0.922
C21:0 (Heneicosanoic)	11.67	13.17	9.60	12.67	1.22	0.098	0.323	0.538
C22:1n9 (Erucic)	13.80 ^B^	31.83 ^A^	12.47 ^B^	17.23 ^B^	1.82	<0.001	0.002	0.007
C22:6n3 (Docosahexaenoic)	31.00	29.07	25.23	32.07	4.11	0.567	0.745	0.317
SFAs ^1^	15,250.40	16,010.40	14,500.87	15,104.27	1968.73	0.738	0.685	0.969
MUFAs ^2^	21,771.97	22,911.40	20,651.17	21,835.53	2436.64	0.646	0.664	0.993
PUFAs ^3^	3310.33	3745.07	3515.30	3394.00	256.10	0.558	0.783	0.309
n-3 PUFAs ^4^	150.77	196.60	155.80	171.80	17.94	0.123	0.597	0.430
n-6 PUFAs ^5^	3045.70	3431.73	3240.47	3109.83	234.68	0.601	0.793	0.303
USFAs	25,082.30	26,656.47	24,166.47	25,229.53	2554.20	0.620	0.659	0.923
TFAs	40,332.70	42,666.87	38,667.33	40,333.80	4466.05	0.666	0.666	0.942

^1^ SFAs (saturated fatty acids) = C10:0 + C12:0 + C14:0 + C15:0 + C16:0 + C17:0+ C18:0 + C20:0 + C21:0; ^2^ MUFAs (monounsaturated fatty acids) = C16:1 + C18:1n9c + C20:1 + C22:1n9 + C24:1; ^3^ PUFAs (polyunsaturated fatty acids) = C18:2n6c + C18:3n3 + C20:2 + C20:3n6 + C20:4n6 + C20:5n3 + C22:6n3; ^4^ n-3 PUFAs = C18:3n3 + C20:5n3 + C22:6n3; ^5^ n-6 PUFAs = C18:2n6c + C20:3n6 + C20:4n6; means within a row followed by different lowercase letters differ significantly (*p* < 0.05) and followed by different uppercase letters differ significantly (*p* < 0.01). *p*-value means the effect of GLY, (VC + NM), or the interaction of GLY and (VC + NM) on the parameter within the same row.

## Data Availability

The original contributions presented in the study are included in the article, and further inquiries can be directed to the corresponding author.
